# A model carbonized papyrus scroll opens a novel path to identifying readable scrolls of the Herculaneum Library

**DOI:** 10.1371/journal.pone.0353485

**Published:** 2026-09-16

**Authors:** Douglas Seiler, Jacob Michael LaManna, Michael McOsker, David Kreimer, Michael Cyrus Daugherty, Jens Dopke

**Affiliations:** 1 UC Berkeley Affiliate, Berkeley, California, United States of America; 2 National Institute of Standards and Technology, Gaithersburg, Maryland, United States of America; 3 Department of Greek and Latin, University College London, Researcher in Papyrology, London, United Kingdom; 4 UC Berkeley, College of Chemistry, Microanalytical Facility, Berkeley, California, United States of America; 5 STFC Rutherford Appleton Laboratory, UK Research and Innovation, Harwell Oxford, England; University of Florida, UNITED STATES OF AMERICA

## Abstract

We report the successful fabrication of a laboratory-made carbonized papyrus scroll and the *virtual* unrolling and reading of its custom leaded ink text, using X-ray tomography. We believe this is the first confirmed proof of concept to show that the presence of lead in Herculaneum scrolls would greatly increase the probability for successful reading. This synthetic model enables systematic variation of ink formulations, including leaded inks, to generate ground-truth datasets for the development and validation of text-detection algorithms intended for use on the Herculaneum scrolls. By altering the ink composition from pure carbon to an ink enriched with lead, an X-ray contrasting agent, our team can provide datasets which can be used to test algorithmic performance. Inks containing only carbon present a more challenging detection scenario, serving as a baseline for recovery efforts. The lab-made scroll was constructed from modern Egyptian papyrus and ink incorporating a soluble lead salt, then carbonized and imaged using a laboratory-grade X-ray tomography system. The scroll was virtually unrolled using custom-developed software. Additionally, we found that a handheld X-ray fluorescence (XRF) device could detect lead in the ink post-carbonization. This suggests that XRF may be a practical screening method for identifying ancient scrolls likely to contain lead-based ink and therefore have a higher probability of being successfully read.

## 1. Introduction

The Herculaneum papyri are a remarkable collection of several hundred ancient papyrus manuscripts discovered in 1752 CE at the Villa of the Papyri in the buried Roman town of Herculaneum, near Naples Italy, next to Pompeii. The villa, likely owned by a member of the Piso family, was unearthed during excavations sponsored by the Bourbon kings of Naples. Hidden beneath layers of volcanic ash and rock since the catastrophic 79 CE eruption of Mount Vesuvius, the scrolls were found carbonized, essentially turned into brittle charcoal, but astonishingly preserved in form, representing the only known intact library from classical antiquity. The overwhelming majority of the texts so far recovered from the Herculaneum library contain philosophical treatises, mostly of the Epicurean school. Most of them were authored by Philodemus of Gadara (ca. 110 BCE to ca. 40 BCE), but important works by Epicurus himself and other Epicureans have been found. A few Stoic texts (apparently all by Chrysippus) and Latin texts (including an epic poem on the Battle of Actium and the recently identified *Histories* of Seneca the Elder) have been found [1]. The importance of the library consists both in the light it sheds on ancient philosophy, not just Epicureanism, but the schools and philosophers against whom they argued, ***but also in the fact that every text in the library is new to us****.* None were transmitted by the usual route of monastic copying through Late Antiquity and the Middle Ages.

The scrolls, tightly rolled and extremely fragile, posed immense challenges to early researchers, who inadvertently destroyed many of them in the process of trying to open them. Still, hundreds were eventually unrolled, though hundreds of scrolls and partial scrolls were left rolled up. Currently almost all the scrolls are in the possession of the Government of Italy and housed in the Biblioteca Nazionale di Napoli.

Each Herculaneum scroll is a series of connected rolled-up sheets of papyrus that were carbonized at high temperatures estimated to range from 400°C to 900°C. A typical Herculaneum scroll is a cylinder with a height of a few dozen centimeters and a diameter on the scale of 10 cm. The length of a fully unrolled scroll varies, with ca. 10 m as a frequently found length. At the time the scrolls were written, carbon black ink was used. This ink was primarily made using carbon obtained from charcoal, soot, or bone black (which was made from charring animal bones in low oxygen). The soot was taken from burning organic products like wood, oil lamps, or resins, which created a fine black pigment. This was mixed with water and a natural binder, usually gum Arabic, a sap from certain types of trees to make ink. Papyrus is a paper-like writing material made from the pith of the papyrus plant, which grows along the Nile. Until recently, reading the still rolled-up Herculaneum scrolls was impossible.

Fortunately, remarkable advancements have been made through AI analysis of data derived from synchrotron X-ray tomography of carbonized scrolls. Most notably, the *Vesuvius Challenge* (https://scrollprize.org), a global competition led by Prof. Brent Seales and Seth Parker, Ph.D., has achieved significant success in virtually reading portions of these ancient texts. As their work progresses, the team continuously refines their AI models and retests their predictive capabilities using high-resolution scans of the scrolls. Our research shows that if lead is present in the ink of a given scroll, it will provide an enhanced imaging dataset that offers teams like the Vesuvius Challenge a superior foundation for further analysis.

A key challenge in using synchrotron X-ray imaging on carbonized scrolls is the poor signal-to-noise ratio (S/N) of the resulting data. This issue arises from the fact that there is virtually no difference in X-ray absorption between regions of papyrus that contain ink and those that do not. Both are composed of carbonized organic material and are nearly identical in their physical and chemical properties [2]. From the standpoint of X-ray absorption and signal generation, the ink is barely distinguishable from the papyrus itself. As a result, the low S/N has long been viewed as an insurmountable obstacle to revealing text within the scrolls.

The discovery of the presence of lead in the letters of two fragments of the Herculaneum scrolls was made using synchrotron-based X-ray fluorescence (XRF) [3], which was further corroborated by analyses of letters, written with black and red inks in other intact ancient papyrus scrolls [4]. Lead is an excellent enhancer of both X-ray fluorescence and absorption. Because lead significantly reduces the transmission of X-ray radiation, its presence can increase image contrast thus effectively improving the signal-to-noise ratio (S/N). The precise mechanism by which lead was introduced into the ink remains under investigation, with possible sources including components of the ink itself or additives used in the ink-drying process [3,5].

We (Seiler – Dopke) hypothesize that, since synchrotron X-ray beams readily penetrate Herculaneum scrolls [2], a custom XRF system could be used to non-invasively identify scrolls that contain lead. These selected scrolls may then yield X-ray tomography datasets with an S/N ratio high enough to enable successful text recovery using current AI-driven methods.

Because direct testing of this hypothesis using original Herculaneum scrolls and synchrotron radiation is challenging due to the fragility of the former and the prohibitive expense of the latter, we limited our efforts to obtain proof-of-concept data using model Herculaneum scrolls.

We used less powerful but readily available instruments for detection of the XRF of lead to evaluate whether this method is suitable for selecting lead-containing scrolls from the Herculaneum Library.

In this study, we describe the construction of a model Herculaneum scroll using modern Egyptian papyrus, inscribed with lead-spiked ink applied via a contemporary stylus. The scroll was then carbonized (Seiler) to closely mimic the physical and chemical properties of ancient Herculaneum scrolls, including the presence of lead in the lettering. Using this model material and a combination of commercial and custom-built XRF and X-ray tomography systems, we evaluated the feasibility of a novel, two-step strategy for reading carbonized scrolls.

The proposed approach involves: (1) in-situ XRF screening of library scrolls to detect the presence of lead in the ink, scrolls with elevated lead content will be selected for synchrotron-based X-ray tomography when beamtime is available, and (2) production of training datasets for algorithm development with known ground-truth by scanning model carbonized scrolls with varying ink compositions using X-ray tomography. We believe that the semi-quantitative results obtained with our model scrolls provide compelling preliminary support for testing this workflow on actual Herculaneum material.

## 2. Materials and methods

### 2.1. Materials

#### 2.1.1. Papyrus.

**Fig 1:**
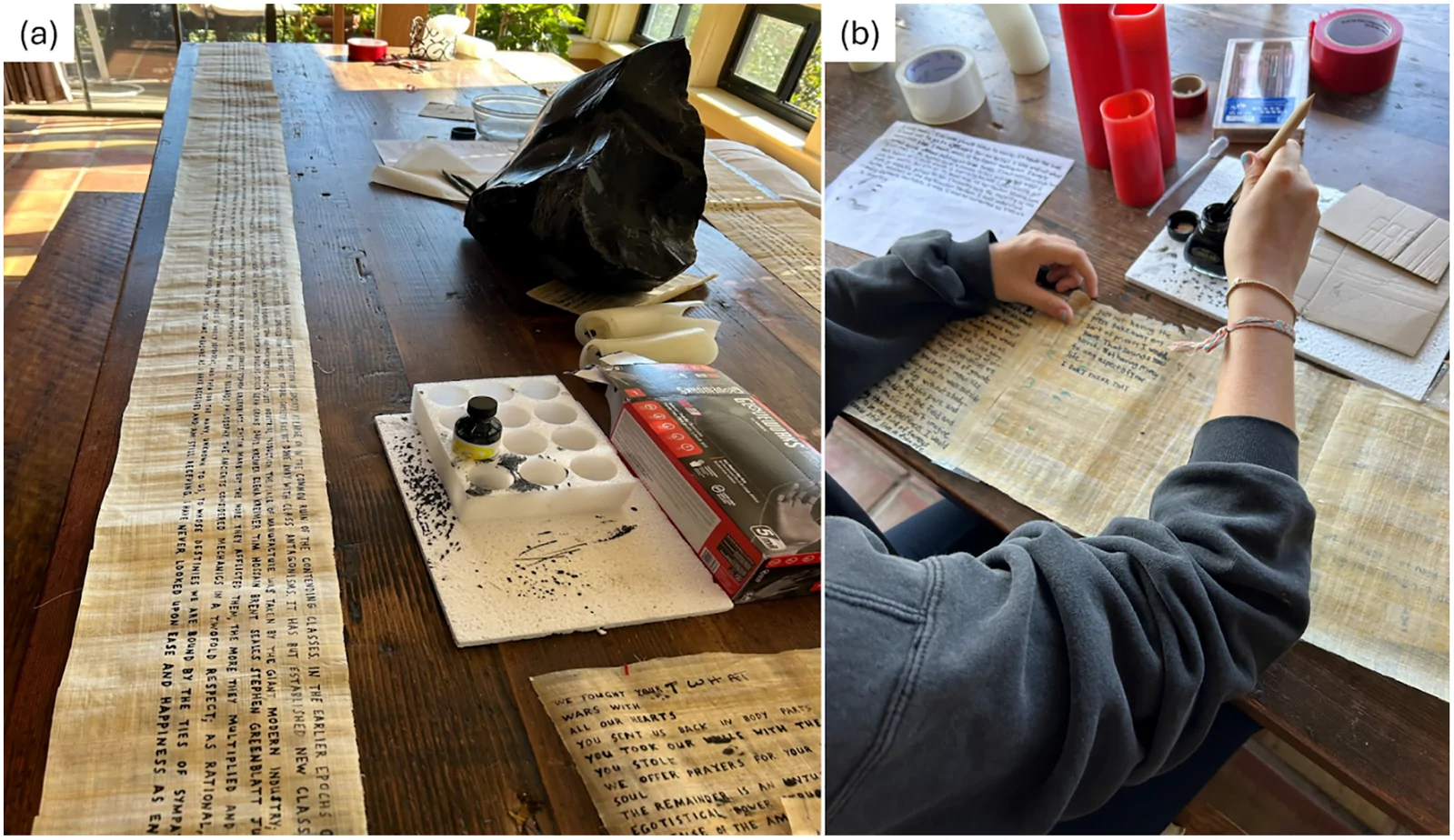
Preparation of papyrus scroll with inks of varying lead concentrations.

Papyrus sheets shown in Fig 1 (10 m length and 0.3 m width; in Scrolls) were manufactured in and purchased from Egypt. The modern papyrus is prepared quite similarly to the papyrus made 2,000 years ago.

#### 2.1.2. Stylus.

The stylus was a reed, a wooden ink pen used to write on papyrus and other materials.

#### 2.1.3. Ink.

The black ink was a water-based pigmented ink “Platinum Carbon Ink” manufactured by a company by the name of Platinum, based in Japan.

### 2.2. ICP-OES analysis of the modern ink for lead content

As expected, the ink did not contain lead in any detectable amount, as was established by ICP-OES (Inductively Coupled Plasma Optical Emission Spectroscopy) analysis using an ICP-OES Optima 7000 DV, Perkin-Elmer at Microanalytical Facility, College of Chemistry, UC Berkeley (Kreimer). The detection limit of the ICP-OES instrument for Pb is 0.04 µg/g in a sample. To prepare a sample, 1 mL of the modern ink was mixed with 1 mL of concentrated nitric acid (HNO_3_) by manually shaking a tube. Then, the 2 mL solution was brought to the final volume of 10 mL with water and filtered twice using a paper filter. An aliquot of so-prepared solution was then analyzed by ICP-OES. Taking into account the dilution of the original ink, the concentration of lead in the liquid component of the modern ink is estimated to be less than 0.4 µg/g.

### 2.3. Preparation of lead-spiked inks

Among common lead salts, lead nitrate (Pb(NO_3_)_2_) has the highest solubility in water, 565 g/L (20°C). The molecular mass of the salt is 331 g/mol, and that for lead is 207 g/mol; one gram of the salt therefore contains 0.625 g of lead.

***Note of Caution***. All lead salts are toxic to humans and animals. Work with lead-salt-containing substances and materials was performed using approved safety protocols.

Lead nitrate purchased from Home Science Tools, Item Number UN1469, was added directly to the Platinum Carbon Ink to prepare a stock solution of lead in ink, containing 360 mg of salt per 1 mL of ink. Alternatively, a lead stock solution of the same concentration of salt was prepared in distilled water. Then, various lead-spiked inks were prepared by combining appropriate amounts of a lead stock solution with the Platinum Carbon Ink to reach the intended final concentrations, (Seiler – Kreimer).

In contrast to simple dilution of the Platinum Carbon Ink with distilled water, the addition of a dry lead nitrate or its solution to the ink, at any concentration relevant to the purposes of this study, caused a noticeable change in consistency. Almost immediately, it became less homogeneous and significantly more viscous and had the consistency of a slightly diluted yogurt. Because the ink is a colloidal solution of charged organic particles (presumably negatively charged), we attribute this change to a known salt-induced colloidal aggregation (coagulation), which is due to salt ion-induced reduction in the repulsive forces between the colloidal particles. The lead-spiked inks, however, did not form a solid residue over time nor did they undergo clearing due to formation of floating particles (at least for a period extending to several weeks). Upon additional dilution with distilled water, the lead-spiked inks generated in this way resulted in fairly well-defined lines and letters, when used for writing on the papyrus.

### 2.4. Equipment for preparation of carbonized papyri

Carbonized papyrus was made by heating papyrus in a semi-sealed steel container in a high temperature furnace with little or no input of oxygen from outside air.

***Note of Caution***. The off-gas was not formally characterized, but could be flammable and toxic, therefore this process was performed mostly under a hood.

### 2.5. X-ray imaging

X-ray imaging was conducted at the National Institute of Standards and Technology (NIST), (LaManna & Daugherty) on the Neutron and X-ray Tomography (NeXT) system [6]. The X-ray system consists of an X-ray Worx 225 kV reflection tube with tungsten anode and a lens-coupled camera detector. The detector converts the X-ray photons to visible light with a 20 µm thick gadolinium oxysulfide scintillator and captures the light with a ZWO ASI6200MM 62-megapixel, 16-bit ADC, air-cooled astro camera with Nikon NIKKOR Z 58 mm f/0.95 S Noct lens set to the minimum focal distance. The lens and camera configuration gave an effective pixel size of 18 µm and a field-of-view of 172.4 mm by 114.9 mm. This detector configuration was used for both radiography and tomography.

Imaging of carbonized papyrus fragments was conducted by mounting the fragment and its cardboard frame directly to the X-ray detector. Five images were taken for each of the fragments, the open beam (no sample), and beam off (camera noise). Each series of five images were median-combined into a single image to reduce the noise level of the image. All image processing for radiography was completed with Fiji [7].

**Fig 2:**
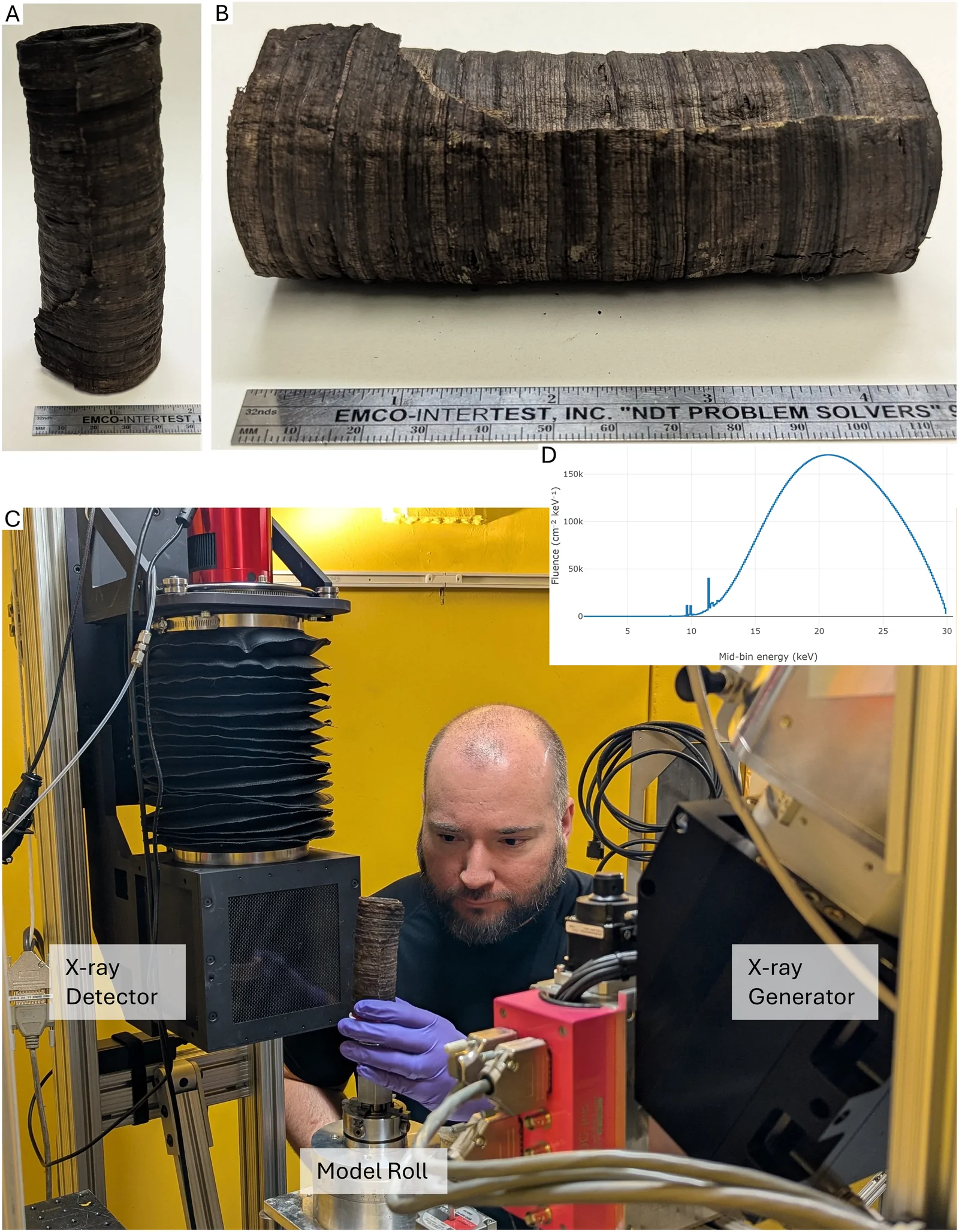
(a) Photo of the model carbonized scroll that was scanned standing upright showing a diameter of 40 mm, (b) model scroll showing length of 110 mm, (c) Jacob LaManna placing the model scroll on the X-ray imaging instrument at NIST, and (d) X-ray spectrum used to image the model carbonized papyrus scroll.

3D tomography was performed by mounting the scroll, shown in Fig 2(a)-2(b), to a rotation stage and spinning the scroll to capture 1001 images from 0° to 360°. The scroll was placed 400 mm from the X-ray tube while the detector was 500 mm from the tube, as shown in Fig 2(c). This positioning was chosen to minimize magnification of the X-ray image on the detector due to the size of the scroll relative to the field-of-view of the detector. A phenolic tube was inserted into the center of the scroll to provide support and mounting to the instrument. The X-ray tube was operated at a maximum voltage of 30 kV and a current of 1.3 mA to increase the signal of the carbon in the scroll. Fig 2(d) shows a plot of the output spectrum of the X-ray tube for these operating setpoints. The spectrum was calculated using SpekPy Web ver 2.0.13.1 [8] with an anode angle of 15°, kqp physics model, Penelope attenuation data, 0.1 energy bins, and a z distance of 500 cm. Each image took 5 seconds to acquire.

Reconstruction of the tomography data was performed with custom code written in Python, (Daugherty) which used the Astra Toolbox [9,10] for the reconstruction. A custom unrolling program was developed to allow the scroll text to be read by tracing splines along the spiral wound scroll and interpolating on normals to the spline.

### 2.6. X-ray fluorescence of papyrus containing writing with lead-spiked ink

**Fig 3:**
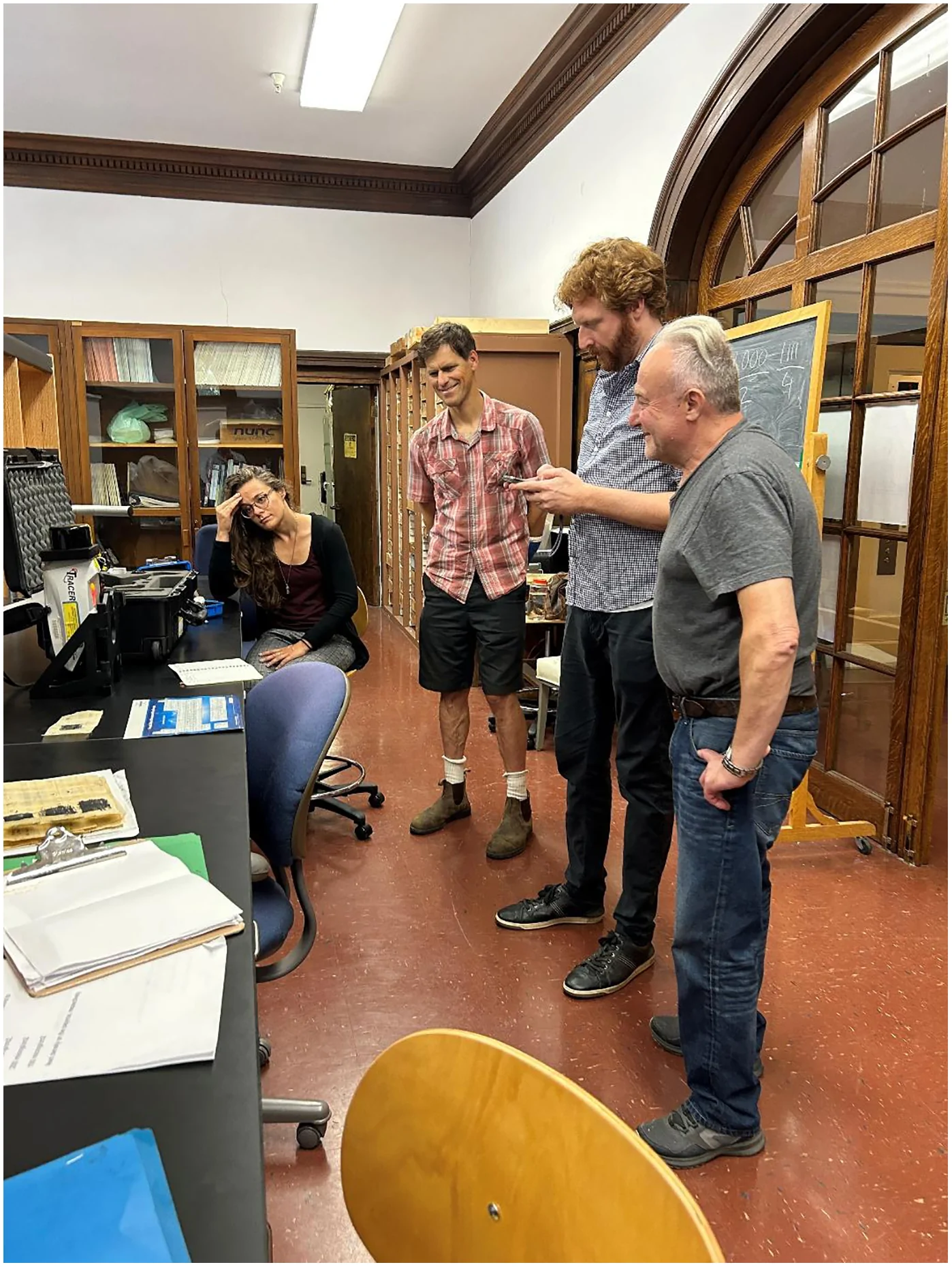
Part of the team conducting XRF on papyrus test pieces at UCB (Left to right: Leah Packard-Gram, Nicholas Tripcevich, Jessy Obert, David Kreimer).

Initial testing of whether X-ray lead fluorescence signals can be detected was performed at UC Berkeley’s Archaeological Research Facility (N. Tripcevich, L. Packard-Grams, & J. Obert) using a Bruker Tracer handheld XRF (See Fig 3). This instrument has a flatbed sample positioning system and therefore only flat fragments of papyrus with written letters were employed to measure back lead XRF signal.

***Image Use.*** Written permission was obtained from N. Tripcevich, L. Packard-Grams and Prof. Karl Van Bibber. While verbal permission to use J. Oberts image was verbal at the time of the photograph, witnessed by co-author David Kreimer.

XRF measurements of the lab-made scroll were carried out at the National Institute of Standards and Technology using an Olympus Innov-X Systems DS-4000CC handheld XRF instrument. This unit was tuned to detect the presence of lead in paint.

### 2.7. Ethics

All authors provided written informed consent for the publication of images of them within this manuscript.

## 3. Results

### 3.1. Building a model of a Herculaneum scroll

First, the material of a modern, commercially available papyrus closely resembles that of ancient scrolls. To generate a model of a Herculaneum scroll, we used a modern carbon black ink, which we believe has a composition similar to inks used in Hellenistic and Roman times. This ink was employed along with a modern version of an ink pen, which is very similar to an ancient stylus.

**Fig 4:**
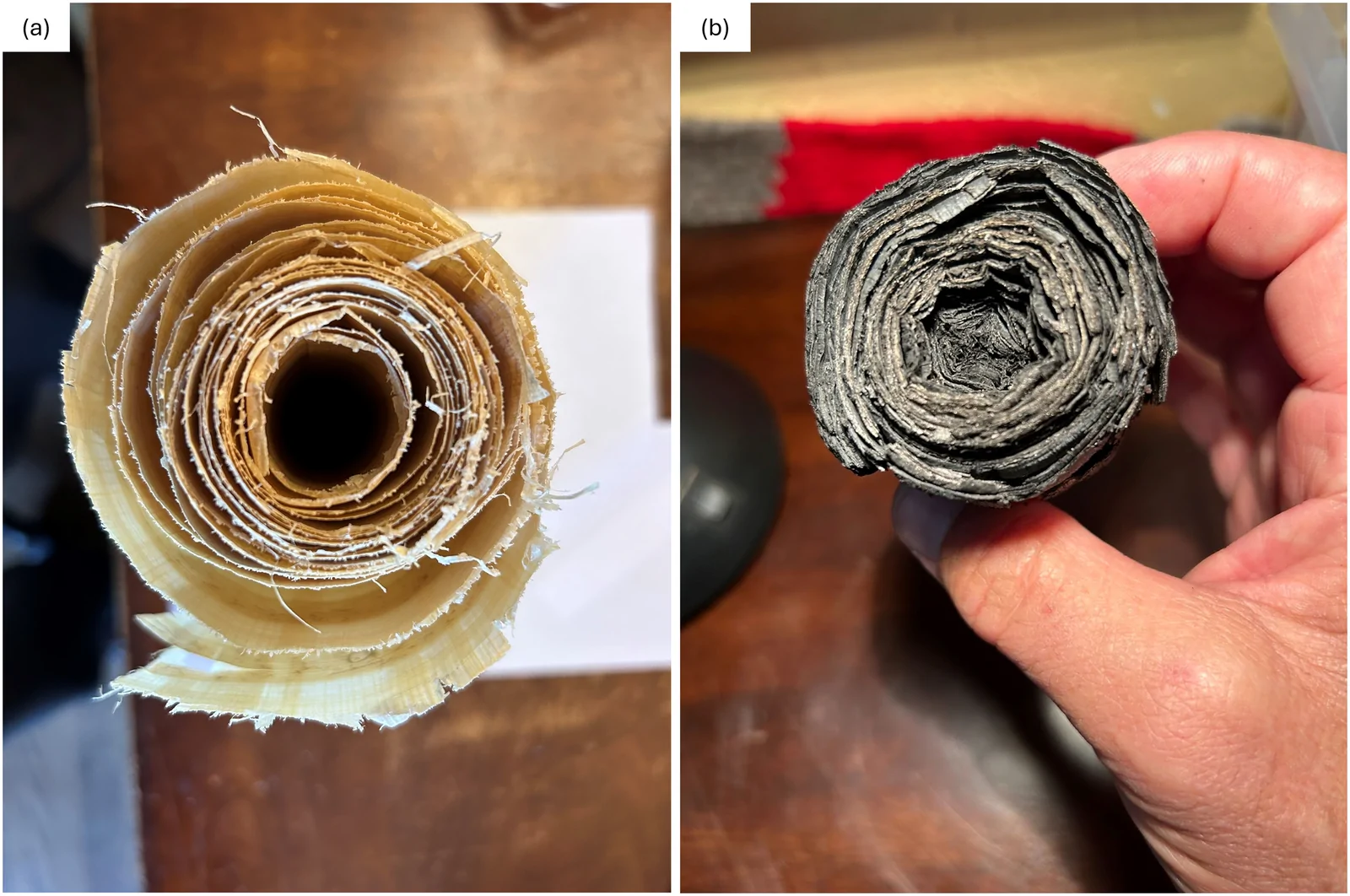
(a) One of several papyrus scrolls before carbonization and (b) after carbonization. Carbonized scroll produced with tight packing of layers and waviness that will challenge unrolling algorithms.

Not surprisingly, writing using these materials (papyrus, ink, and stylus) was very comfortable and produced excellent results (see an example in Fig 1). Second, upon carbonization, scrolls generated in our laboratory (Fig 4) closely resembled some Herculaneum scrolls.

Similar to the scrolls from the Herculaneum Library, model scrolls are extremely brittle, and any attempt to unroll them resulted in fragmentation.

### 3.2. Generation of lead-spiked inks with varying lead concentration

The presence of lead in the ink of two Herculaneum papyrus fragments was demonstrated using synchrotron-based XRF [3]. The study estimated the mass concentration of lead in the letters to be 84 µg/cm^2^ ± 5 µg/cm^2^ for the larger sample, and 16 µg/cm^2^ ± 5 µg/cm^2^ for the smaller sample. In addition, a recent detailed synchrotron-based XRF analysis of fragments of ancient Egyptian papyri from the Roman period (circa 100 CE to 200 CE) [4], which were presumably written in a similar manner to the somewhat earlier Herculaneum scrolls, also confirmed the presence of lead in letters written in red and black inks. According to this analysis, the origin of lead compounds detected in the ink remains unclear: the lead compounds might come from a drying agent rather than from the ink itself [5].

Regardless of the lead’s origin, these findings strongly suggest that letters in the scrolls of the Herculaneum Library may contain lead. This lead can produce an XRF signal that would be detectable by a commercial XRF instrument and thus help select appropriate Herculaneum scrolls for further synchrotron X-ray tomography and analysis.

To experimentally evaluate whether the X-ray signal from lead-containing ink is detectable, it is essential to establish conditions that produce letters with lead concentrations comparable to those observed in ancient samples. One approach is to use the reported surface concentrations of lead in ancient writing as a benchmark, and to formulate a model lead-spiked ink that achieves similar surface concentrations upon application. Since the drying process of lead-spiked ink results in the retention of nearly all organic and inorganic components on the papyrus surface at points of stylus contact, the task reduces to determining the volume of ink required to cover a known area. With this measurement, the appropriate quantity of lead salt can be calculated and incorporated into the ink formulation, enabling a direct conversion between the desired surface concentration of lead and the corresponding concentration within the ink volume.

**Fig 5:**
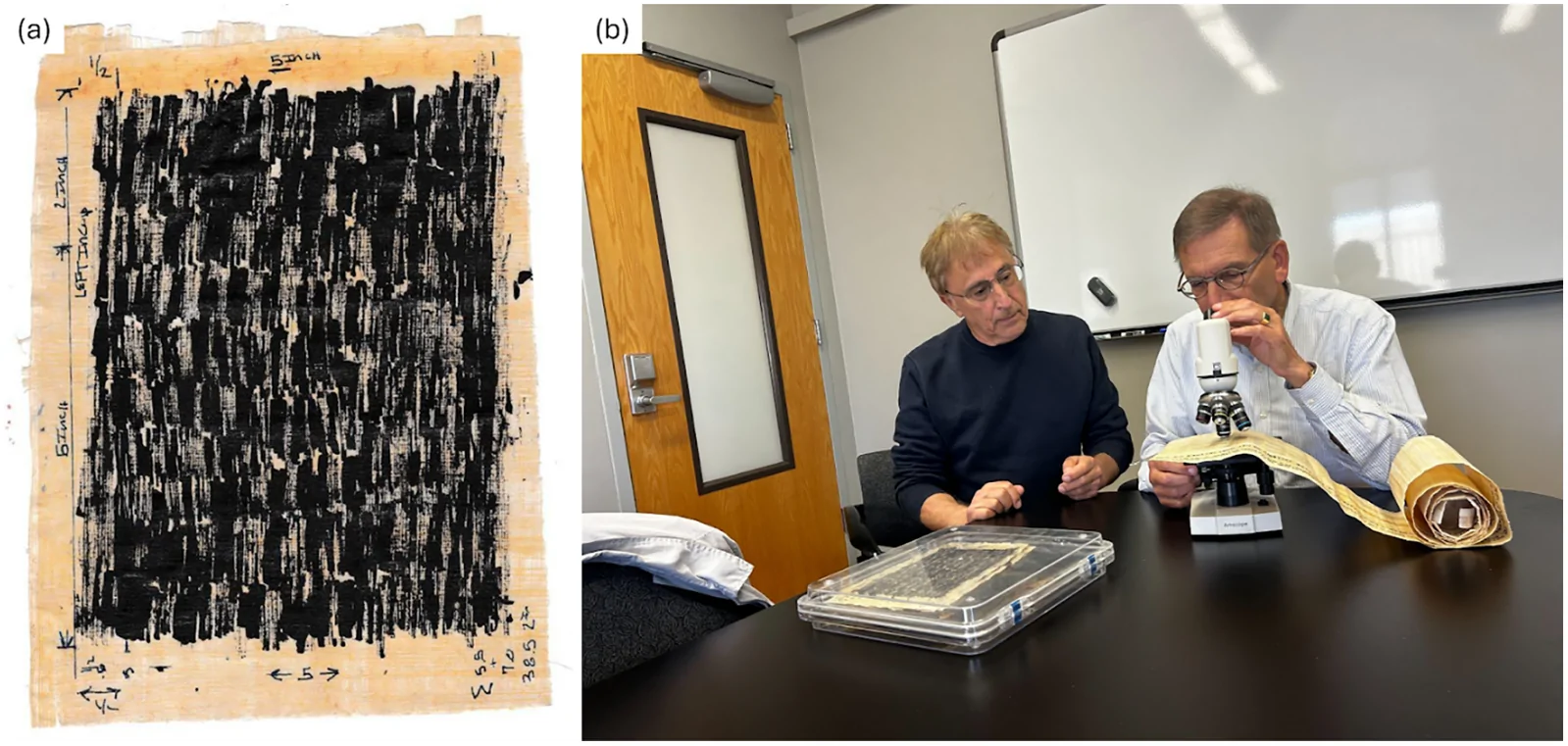
(a) Papyrus used to calibrate ink areal density. (b) Douglas Seiler and Prof. Karl van Bibber, Nuclear Engineering Dept., at UCB, checking ink density.

Inspired by the famous “Black Square” painted by Kazimir Malevich with oil on a canvas (1915), we covered a square (of arbitrary size, 15 cm by 15 cm, i.e., 225 cm^2^) on a model papyrus with stylus-written lines so that there was little to no space between the lines. It turned out that covering almost the entire surface, as shown in Fig 5, required a surprisingly small amount of the model ink, about 0.4 g to 0.5 g, whether spiked with lead or not. This was determined by measuring the mass of an ink-containing flask before and after drawing the square.

Since the ink is predominantly water, we approximate its density to be 1.0 g/cm^3^. Based on experimental coverage, we found that 0.4 to 0.5 mL of ink is sufficient to write over 225 cm^2^ of papyrus. This implies that only about 2 µL of ink is needed to cover 1 cm^2^ with lines. Consequently, the total amount of lead present in 2 µL of lead-spiked ink will determine the surface concentration of lead on 1 cm^2^ of the papyrus.

To replicate the surface concentrations of lead reported by Brun et al. in their analysis of a large Herculaneum fragment [3], we formulated an ink containing 100 µg of elemental lead per 2 µL, which is equivalent to 50 g/L. This concentration yields lead surface densities consistent with those observed in authentic scroll fragments, making the ink formulation suitable for producing modern analogues with comparable X-ray visibility.

As the ink is mostly water, its density (for the purposes of our study) can be assumed to be roughly equal to the density of water, 1.0 g/cm^3^, and thus we deduced that only 0.4 mL to 0.5 mL of ink is required to cover 225 cm^2^. This means that to cover 1 cm^2^ with lines, only about a 2 microliter volume of ink is needed. In turn, whatever amount of lead would be present in this small volume of lead-spiked ink would end up spread over 1 cm^2^ of a line-covered area of papyrus. Then, if 2 microliters of the lead-spiked ink contained lead in the amount of approximately 100 micrograms, writing with it would produce letters containing lead at a surface concentration approximately equivalent to that reported by Brun et al. for their larger fragment [3]. Thus, spiking lead into the ink to the final concentration of elemental lead, by mass, of 100 microgram per 2 microliters (or 50 g/L) results in lead-spiked inks suitable for producing lines similar to those of the larger sample of Herculaneum scrolls of the Brun *et al.* study [3].

### 3.3. Lead X-ray fluorescence signal from letters written with a lead-spiked ink can be detected with a commercial instrument

Several samples were prepared of papyrus (4 cm by 4 cm) fragments containing a 1 cm tall letter written with an ink spiked with lead yielding final lead concentration in the letter on the papyrus of 200 µg/cm^2^, 100 µg/cm^2^, 50 µg/cm^2^, 20 µg/cm^2^, and one control sample containing 0 µg/cm^2^ of lead (prepared using unspiked ink). As expected from ICP-OES analysis of the ink for lead content, which showed that the ink does not contain lead down to the detection limit of the method. In testing with a Bruker Tracer handheld XRF unit supplied by Nate Davies at Bruker, the control sample did not produce any detectable signal. However, all other samples generated a reliably detectable XRF signal, which declined as the lead concentration decreased.

### 3.4. Reading of a model scroll using X-ray tomography image analysis

**Fig 6:**
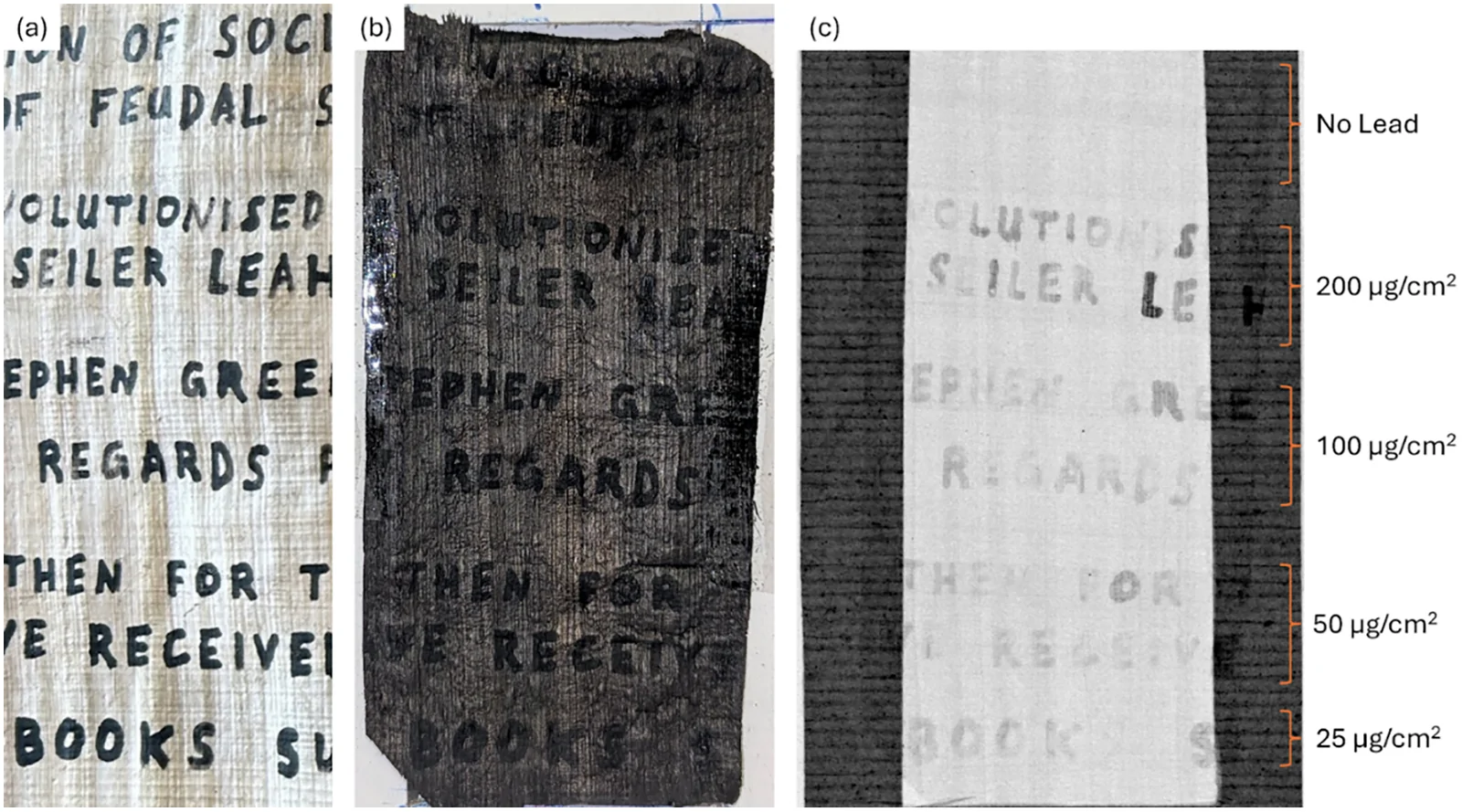
Determination of X-ray sensitivity to ink lead concentration on papyrus. (a) “Ground truth” optical image pre-carbonization, (b) optical image post-carbonization, and (c) X-ray transmission image.

Initial X-ray imaging experiments were conducted using small fragments of carbonized papyrus bearing text written in lead-spiked ink. These tests systematically varied the concentration of lead line-by-line to determine detection limits, as illustrated in Fig 6. In this example, the top two lines contained no lead, while the next two lines were written with ink containing 200 µg/cm^2^ of lead. Subsequent lines contained progressively lower concentrations, down to 25 µg/cm^2^.

The image was processed using flat-field correction and dark-current subtraction, and contrast was adjusted to enhance text visibility. No further enhancement or text-recognition algorithms were applied [11,12]. As expected, the two lines written without lead were not visible in the 2D X-ray image due to the lack of contrast between the carbon-based ink and the carbonized papyrus substrate. In contrast, lines containing lead were clearly resolved, with the 200 µg/cm^2^ concentration providing the highest contrast. Notably, even the lowest concentration tested, 25 µg/cm^2^, was detectable.

While 2D radiography relies primarily on material contrast, moving to 3D tomography may allow for detection of lead-free ink through structural resolution, potentially revealing text based on geometry rather than composition alone.

**Fig 7:**
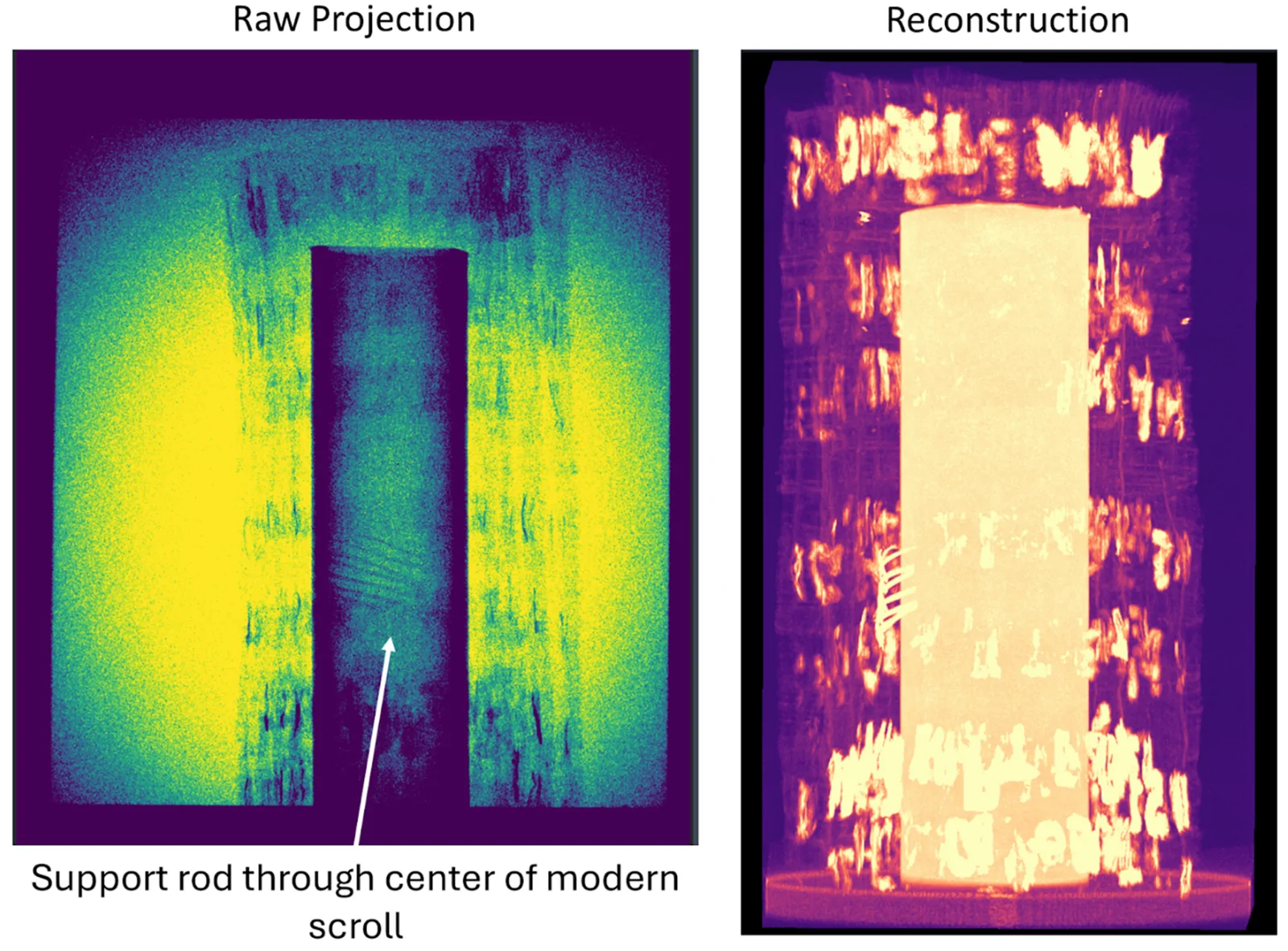
Raw projection image of model carbonized scroll showing indications of lead ink and reconstructed volume with contrast adjusted to show lead ink.

After initial X-ray imaging of the papyrus fragments, efforts moved to performing X-ray tomography on the model carbonized scroll. Initial X-ray images (raw projections) of the scroll showed obvious indications of the lead-containing ink as shown in Fig 7.

**Fig 8:**
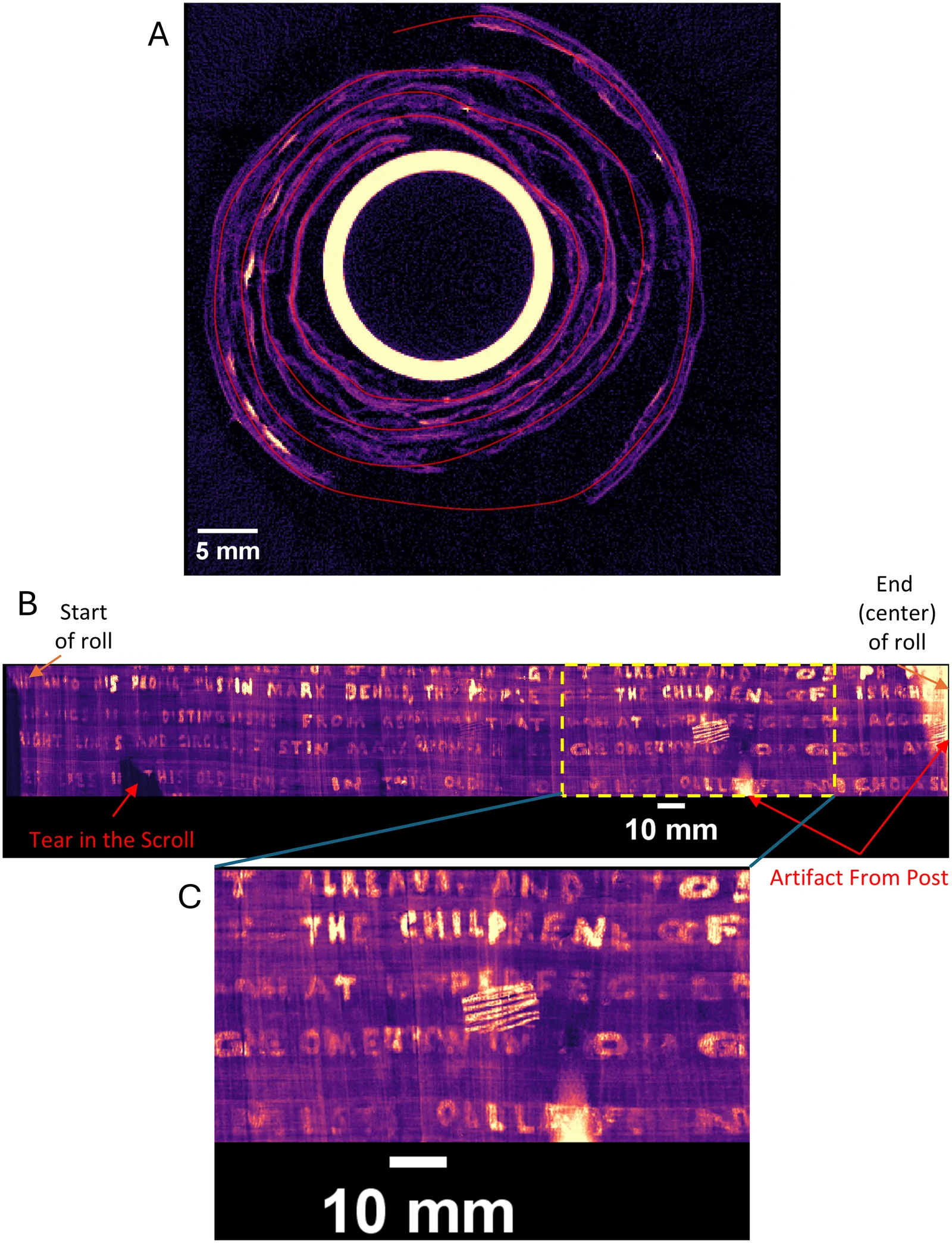
(a) unrolling program showing drawn splines following the curvature of the scroll, (b) full virtual unrolling of model scroll, (c) highlighted visible text from (b).

Once the data was processed and reconstructed to form the 3D data volume, the letters became apparent but not possible to read or decipher. The unrolling program required splines to be placed along the rough center of the spiral of the scroll at multiple points along the length. This produced the map to convert the spiral to a flat plane. Selecting an appropriate point in the unrolled scroll allows the text to be seen as shown in Fig 8(a). The text shown in Fig 8(b) is reconstructed with no additional processing to enhance or extract. It may be possible to automate enhancement and extraction of the text with additional algorithms beyond what is demonstrated here. Without any enhancement, it is possible to clearly see “the children of” in the zoomed-in region in Fig 8(c).

**Fig 9:**
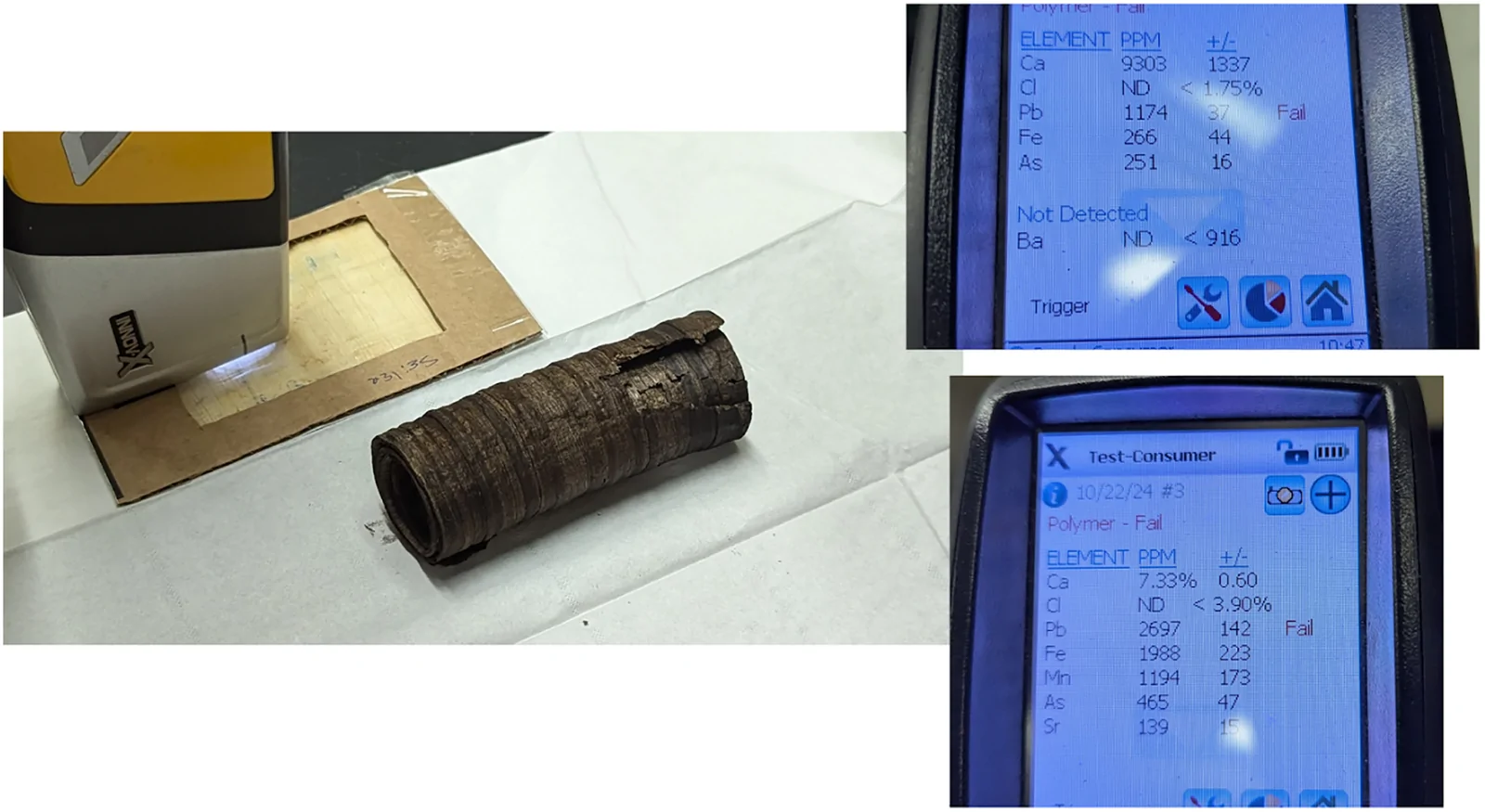
XRF testing of imaged model scroll showing multiple measurements that detected the presence of lead.

After the success in seeing the letters on the scroll with X-ray imaging, the scroll was taken for XRF testing at NIST using the Olympus handheld XRF. Several positions along the length of the scroll were tested for the presence of lead. The handheld XRF was held above the scroll in near contact but not touching its surface. As shown in Fig 9, lead was detected on the scroll. Additional effort is required to develop a more efficient or automated way to scan scrolls with a handheld unit.

## 4. Discussion

One of the major efforts of research into the Herculaneum Library has been to somehow extract the texts that are trapped within these brittle, precious pieces of carbonized organic matter. Initially, to develop a method for unwrapping the carbonized Herculaneum scrolls, one of us (Seiler) designed a model of such a scroll. The model employs modern materials and tools similar to those used by the creators of the original scrolls: papyrus, carbon ink, and a stylus. Further, carbonization-causing procedures were devised that may mimic those of the Vesuvius eruption. The resulting model scrolls closely resemble those found in the Villa (Fig 4). Model scrolls were produced in our laboratory at UC Berkeley in numbers sufficient for experimentation.

**Fig 10:**
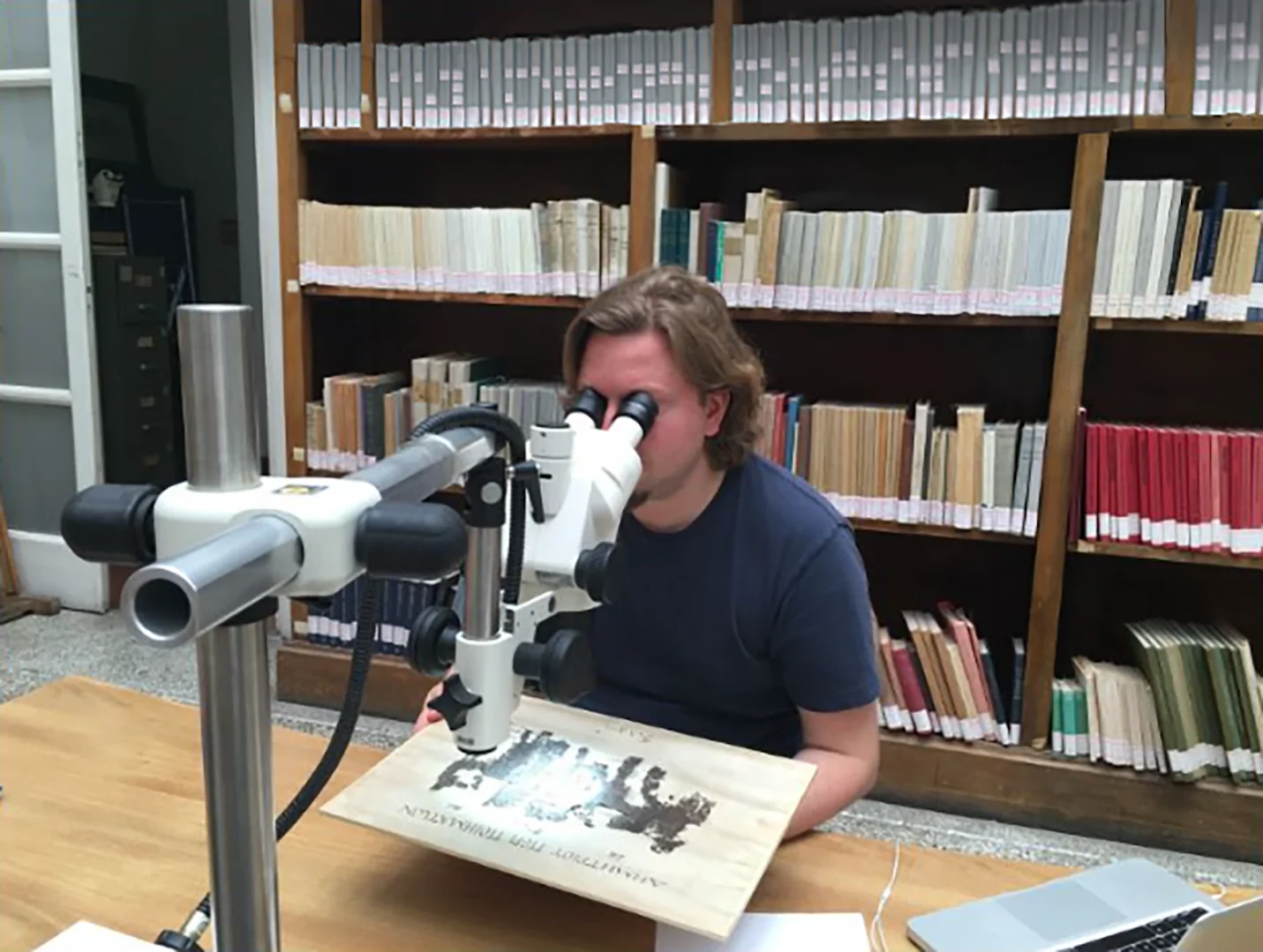
Michael McOsker examining a previously unrolled Herculaneum papyrus (PHer. 1012 Demetris Laco, On Poems ll).

X-ray radiation can pass through various materials non-destructively and can be used to visualize features hidden inside a scroll compared to optical methods that require the scroll to be opened (Fig 10). The basis for this visualization is that X-rays are more readily absorbed as atomic number increases, thus materials with greater electron density provide greater contrast. A cross-section of the passed radiation therefore displays areas of higher and lower intensity reflecting the difference and localization of hidden features.

Particularly successful acquisition of such X-ray absorption images was accomplished using a synchrotron. Recently, when this well-established method of data collection was combined with the power of machine learning algorithm-guided building of images, one of the Herculaneum scrolls could be partially read, and fragments of text in another were obtained as well. See Vesuvius Challenge Winners | Vesuvius Challenge (https://scrollprize.org/winners.)

The use of model scrolls provides a path forward to developing algorithms to read scrolls with poor S/N by providing ground truth with known text. These scrolls can be created with varying formulations and thicknesses of ink, writing styles and languages, carbonized, and scanned with X-ray tomography to provide training datasets. Since that text was initially written on the scroll before carbonization is known, it would be possible to score algorithms on their ability to recover the text. This process can be performed in a blinded manner where the algorithm developers/scroll readers are not given the solution during the reading process. Once robust algorithms are developed and trained on scrolls with lower signal to noise, it would be possible to apply to the real Herculaneum scrolls.

Fortunately, at least some of the written elements of intact ancient scripts, contemporary to Herculaneum scrolls, were discovered to contain non-organic matter, including lead. While algorithms are being developed with the model scrolls, it would be possible to use a tuned handheld XRF to identify candidate Herculaneum scrolls containing lead signals for tomography analysis at a synchrotron facility. This would provide a means of identifying scrolls that could provide higher likelihood of being read immediately. To make this process as efficient as possible, a scanning methodology would need to be developed to efficiently scan many scrolls with high confidence of lead detection.

## 5. Conclusions

In this work, we developed a method to create model carbonized scrolls with ink of varying composition of lead in the ink. Carbon-based inks were mixed with lead nitrate to produce lead-spiked inks to recreate lead levels found in ancient texts. This ink was used to write text on modern Egyptian papyrus to create papyrus scrolls that were then carbonized in a low-oxygen environment. These carbonized papyrus scrolls showed similar density to the Herculaneum papyri. X-ray imaging and XRF were used to determine if the lead was detectable and whether the text could be recovered without physically unrolling the carbonized scroll. We found that we could detect text on the model scroll with lead levels down to 25 µg/cm^2^, which was the lowest level tested. A handheld XRF device tuned for lead detection was also able to detect the presence of lead on the model scroll.

The findings here present a path forward for developing algorithms to identify Herculaneum scrolls that contain lead and have the highest chances of being read in a shorter timeframe and provide a technique for development of algorithms for the more challenging scrolls. There has been recent success in reading these rolls, but they still present problems [13]. A tuned XRF device could be used to sort scrolls and determine ones with lead content before they are shipped to synchrotron facilities. Model scrolls can be produced with various ink compositions, with text known before carbonization which provides ground truth to test algorithms for text identification. This provides a low-risk method to develop algorithms without endangering the priceless artifacts.
